# A Review on Anti-Inflammatory Activity of Monoterpenes

**DOI:** 10.3390/molecules18011227

**Published:** 2013-01-18

**Authors:** Rita de Cássia da Silveira e Sá, Luciana Nalone Andrade, Damião Pergentino de Sousa

**Affiliations:** 1 Department of Physiology and Pathology, Federal University of Paraíba, CP 5009, CEP 58051-970, João Pessoa, Paraíba, Brazil; 2 Department of Pharmacy, Federal University of Sergipe, CEP 49100-000, São Cristóvão, Sergipe, Brazil

**Keywords:** monoterpenes, essential oils, natural products, medicinal plants, anti-inflammatory activity, inflammation, cytokines, immunomodulatory activity, asthma, allergy

## Abstract

Faced with the need to find new anti-inflammatory agents, great effort has been expended on the development of drugs for the treatment of inflammation. This disorder reduces the quality of life and overall average productivity, causing huge financial losses. In this review the anti-inflammatory activity of 32 bioactive monoterpenes found in essential oils is discussed. The data demonstrate the pharmacological potential of this group of natural chemicals to act as anti-inflammatory drugs.

## 1. Introduction

Inflammation is a complex biological response of vascular tissues against aggressive agents such as pathogens, irritants, or damaged cells. It can be classified as either acute or chronic, and involves a cascade of biochemical events comprising the local vascular system, the immune system, and different cell types found in the injured tissue. Acute inflammation is the initial response and is characterized by the increased movement of plasma and innate immune system cells, such as neutrophils and macrophages, from the blood into the injured tissues. Chronic inflammation concerns a progressive change in the type of cells present at the site of the inflammatory reaction and is characterized by simultaneous destruction and healing of the injured tissue [1].

Regardless of the triggering factor, the mechanisms involved in the inflammatory process are common to all and the standard signs of inflammation are expressed by increased blood flow, elevated cellular metabolism, vasodilatation, release of soluble mediators, extravasation of fluids and cellular influx [1]. Upon the presence of the inflammatory agent, cell membranes induce the activation of phospholipase A_2_ followed by release of arachidonic acid and inflammatory mediators such as cytokines, serotonin, histamine, prostaglandin and leukotrienes that increase vascular permeability, thus facilitating the migration of leukocytes to the site of inflammation [2,3].

A closer look at this process shows that during the initial stages of inflammation, leukotrienes, prostaglandins, and histamine bind to their receptors on endothelial cells leading to vasodilatation, contraction of endothelial cells, and increased blood vessel permeability. The binding of histamine sets off an upregulation of P-selectin (a cell adhesion molecule) and platelet-activating factor (PAF) on the endothelial cells that line the venules. The subsequent binding of P-selectin and PAF to leukocytes leads to extravasation followed by leukocyte migration towards chemotactic agents (e.g., complement protein C5a and leukotriene B4) produced by cells at the site of injury. In addition, activated macrophages and vascular endothelial cells release inflammatory cytokines such as tumor necrosis factor (TNF) and interleukin-1 (IL-1) that bind to receptors on endothelial cells maintaining the inflammatory response by upregulation of the production of the adhesion molecule E-selectin and keeping the expression of P-selectin. E-selectin binds to leukocytes, which, then, move across the basement membrane towards chemokines such as interleukin-8 (IL-8) and monocyte chemotactic protein-1 (MCP-1) generated by cells at the infected site [1,2,3].

Inflammation is a protective measure taken by the organism to eliminate the injurious stimuli; however the use of anti-inflammatory substances can be an effective tool in the therapeutic treatment of the diseases. In this context, medicinal plants and their isolated compounds are employed worldwide in folk medicine to treat different inflammatory conditions, such as lung and skin inflammations. In the continuous search for new bioactive natural products against inflammation, essential oils are increasingly being referred to as a rich source of such products, therefore the anti-inflammatory activity of some essential oils and their isolated components, *i.e.*, the monoterpenes, is reported below. Most investigations concerning the anti-inflammatory potential of agents are undertaken in rodents with the use of different test models such as the carrageenan, formalin and arachidonic acid induced paw edema, among others, and *in vitro* and *ex vivo* protocols, which may employ essential oils or monoterpenes.

Essential oils of aromatic plants such as *Helichrysum odoratissimum* (L.) Less., *Heteropyxis natalensis* Harv. and *Lippia javanica* (Burm. f.) Spreng., species popularly employed in the treatment of dermatological pathologies, were used in experimental assays to determine the 5-lipoxygenase inhibitory activity of these species. This enzyme is a member of the lipoxygenase family that converts essential fatty acids into leukotrienes, proinflammatory mediators which are mainly released from myeloid cells. The essential oils of these aromatic plants and two of their major components, namely 1,8-cineole and limonene, are believed to contribute to the anti-inflammatory activity of these species, in which 1,8-cineole has been shown to cause a partial potentiation of the anti-inflammatory action exhibited by limonene [4]. In another study, the investigation of the immunostimulating activity of essential oils of various plants (lemon, eucalyptus, marjoram, juniper, thyme, lavender, mentha, rosemary, geranium, pine, and salvia) and the monoterpenes 1,8-cineole, menthol, citral, α-pinene, limonene, linalool, thymol, camphor, and borneol showed that the pine and lemon oils had the strongest immunostimulating activity, while α-pinene displayed the strongest action among the monoterpenes, followed by borneol and 1,8-cineole [5]. With a similar purpose, Standen and collaborators [6] tested *in vitro* a selection of essential oils obtained from *Matricaria recutita*, *Boswellia carteri*, *Pelargonium graveolens*, *Lavandula angustifolia*, *Citrus limon*, *Melaleuca alternifolia*, *Melaleuca viridiflora*, *Santalum spicatum*, *Cedrus atlantica* and *Thymus vulgaris*, and the monoterpenes α-pinene, (*S*)-(−)-limonene, linalool, geraniol, thymol, 1,8-cineole, linalyl acetate, and (+)-terpinen-4-ol for potential immunomodulating effects on natural killer cell activity (NKCA) and lymphocyte activation through CD69 expression. Linalyl acetate was the only monoterpene that exhibited a dose-dependent stimulation of NKCA, whereas no other effects of essential oils or the other monoterpenes were found.

The evaluation of the immunosensitizing potential of monoterpenes present in the essential oils of a variety of aromatic, edible and medicinal plants was performed in the rat popliteal lymph node assay (PLNA), which is also a test that has also been proposed as a screening tool for detecting chemicals with potential of inducing allergic and auto-immune-like reactions in humans. PLNA was positive for the monoterpenes citral, α-terpinene, β-myrcene and (−)-α-pinene, and negative for (−)-menthol, 1,8-cineole, (±)-citronellal, (+)-limonene, (±)-camphor and terpineol. A secondary PLNA, a T-cell priming test, with the four substances tested positive in the primary assay showed that citral, α-terpinene, β-myrcene and (−)-α-pinene were negative in the secondary assay, indicating that these monoterpenes induced a immunostimulatory response due to their irritant properties, but no monoterpene seemed to be a sensitizing agent in the PLNA [7]. Furthermore, the investigation of the inhibitory action of essential oils obtained from various plants against the production of leukotriene as a mediator of inflammation showed that at least a compound selected from a group consisting of (−)-menthol, (+)-limonene, α-terpinene, γ-terpinene, terpineol, β-myrcene, (±)-linalool, geraniol, citral, α-cyclocitral, (+)-α-pinene, (−)-α-pinene, and (+)-*cis*-verbenol was effective as an inhibitor of leukotriene production, indicating that the inhibitor can be used for a variety of inflammatory diseases, including asthma, chronic bronchitis, and allergic rhinitis, among others [8]. The investigation of the antiasthmatic properties of the essential oil extracted from the leaves of *Artemisia argyi*, and its monoterpenic constituents *trans*-carveol, α-terpineol/l-α-terpineol, α-phellandrene, camphene, borneol acetate, isoborneol, and carvone besides the sesquiterpenes elemol and α-cedrene in guinea pigs indicated that α-terpineol/l-α-terpineol and *trans*-carveol were antiasthmatic and that α-terpineol was more effective than the essential oil [9,10]. In another study, the constituents of *Ocimum basilicum* Benth essential oil such as estragole, 1,8-cineole, ocimene, linalool acetate, 1-epibicyclosesquiphellandrene, menthol, menthone, cyclohexanol, cyclohexanone, myrcenol and nerol were also tested and only myrcenol and nerol exhibited antiasthmatic activity [11].

Essential oils are a mixture of volatile and natural substances, characterized by a strong odor and produced by aromatic plants as secondary metabolites. They have a wide range of applications and have been commercially important for the pharmaceutical, food, cosmetic and perfume industries. The variety of pharmacological activities found in essential oils is remarkable. This class of natural products is attracting the interest of many researchers to investigate its potential as drugs for the treatment of various diseases. The number of clinical and pre-clinical studies about essential oils and their chemical constituents is increasing every year. Furthermore, there are many bioactive substances that are synthesized from constituents of essential oils. Some pharmacological activities of these oils, such as antitumoral and antinociceptive actions are related to their anti-inflammatory effects [12,13,14,15,16] The monoterpenes are natural products belonging to the chemical group of terpenes and the main constituents of essential oils. They are found in many bioactive essential oils and medicinal plants [12]. Considering that the monoterpenes are common in many plant species and are used in cosmetic and pharmaceutical preparations, as well as in the food industry, it is important to review the pharmacological potential of monoterpenes with the anti-inflammatory activity.

## 2. Methodology

The present study was carried out based on the literature review of the monoterpenes from essential oils with anti-inflammatory activity. Information about 32 bioactive compounds is given in this article.

Chemical structure and name of bioactive compounds, as well as the corresponding references are provided. The monoterpenes presented in this review were selected on the base of the effects shown in specific experimental models for evaluation of the anti-inflammatory activity and/or by complementary studies, which have aimed to elucidate the mechanisms of action. To select the essential oils constituents terms related to the theme, such as “essential oils” and “monoterpenes”, were used, as well as names of representative compounds of these chemical groups refining with “asthma, antiasthmatic effect, allergy, antiallergic effect, inflammation, anti-inflammatory, immune response, lymphocytes, cytokines, immunoglobulins or immunoregulatory”. A search was performed in the scientific literature databases Chemical Abstracts until January 2012.

## 3. Results and Discussion

### 3.1. 1,8-Cineol, l-Menthol, Menthone, and Neomenthol

Cineole or 1,8-cineole or 1,8-cineol (eucalyptol) is a monoterpene oxide present in the essential oil of many plants, for example eucalyptus, popularly used to treat respiratory diseases aggravated by infection [17]. The anti-inflammatory activity of 1,8-cineole was investigated by using the trinitrobenzenesulfonic acid (TNBS)-induced colitis model in rats, which is one of the most common experimental models used in screening drugs active against human inflammatory bowel disease [18]. Male Wistar rats received 1,8-cineole (200 and 400 mg/kg) rectally, 24 and two hours before (pre-treatment) or two and 24 h after (post-treatment) the induction of colitis via intracolonic administration of TNBS. The administration of TNBS induced an extensive inflammation and ulceration in the colon associated with an increase in myeloperoxidase (MPO) activity, an indicator of neutrophilic infiltration. Myeloperoxidase is an enzyme released from storage granules following activation of the neutrophils by inflammatory stimuli, showing potent proinflammatory properties and a direct contribution to tissue injury [19]. Animals pre-treated but not post-treated with 1,8-cineole showed a significant reduction in gross damage scores and wet weights of the inflamed colonic tissue, a parameter considered a reliable and sensitive indicator of the severity and extent of the inflammatory response [20]. In addition, it also significantly reduced MPO activity, thus indicating anti-inflammatory effect of 1,8-cineole with a possible preventive action for gastrointestinal inflammation and ulceration [17].

The anti-inflammatory action of 1,8-cineole was also demonstrated in the Wistar rat submitted to the carrageenan-induced paw edema and cotton pellet-induced granuloma models. The treatment with 1,8-cineole (100, 200 and 400 mg/kg, p.o.) caused a significant reduction in paw edema by 26%, 26% and 46%, respectively, although the standard drug, indomethacin (5 mg/kg, p.o.), showed a greater inhibition (62%). The effects of 1,8-cineole and indomethacin on cotton pellet-induced granuloma also indicated anti-inflammatory action of these substances as seven days after treatment, both the wet and dry weights of granulation tissue surrounding the pellets were significantly reduced in the 1,8-cineole (400 mg/kg) treated group by 37% and 40%, respectively, and in the indomethacin group (5 mg/kg) by 25% and 55%, respectively [13].

In the humans, a clinically relevant anti-inflammatory action of 1,8-cineole in bronchial asthma was reported in a double-blind placebo-controlled trial that evidenced a mucolytic and steroid-saving effect of 1,8-cineole in bronchial asthma patients [21]. A previous study by this author reported the inhibitory effect of 1,8-cineole on lipopolysaccharide (LPS)-and interleukin (IL)-1β (IL-1β)-stimulated mediator production by human monocytes *in vitro*, showing a decreased production of tumor necrosis factor α (TNF-α), IL-1β, leukotriene B4 (LTB4) and tromboxane B2, an indication that 1,8-cineole could be useful in long term treatment of airway inflammation in bronchial asthma and other steroid-sensitive disorders [22]. Moreover, the evaluation of the effect of 1,8-cineole on arachidonic acid metabolism in blood monocytes of asthma patients revealed that the production of LTB4 and prostagladin E2 (PGE2) measured from isolated monocytes was significantly inhibited in patients (−40.3% and −31.3%, respectively) and health subjects (−57.9% and −41.7%, respectively). Similarly, the effect of 1,8-cineole (1.5 μg/mL) on monocyte mediator production was studied *in vitro* and compared to the effects of inhaled budesonide (108 M), a glucocorticoid steroid used for the treatment of asthma and non-infectious rhinitis. Both 1,8-cineole and budesonide exerted similar inhibitory effects as evidenced by the reduced *in vitro* production of LTB4 (−27.9%, −23%), PGE2 (−75.5%, −44%), and IL-1β (−84.2%, −52%) [22].

Juergens and collaborators [23] also investigated the anti-inflammatory efficacy of 1,8-cineole in inhibiting polyclonal stimulated cytokine production by human unselected lymphocytes and LPS-stimulated monocytes. At 1.5 μg/mL, 1,8-cineole significantly inhibited cytokine production in lymphocytes of TNF-α (92%), IL-1β (84%), IL-4 (70%) and IL-5 (65%), and monocytes of TNF-α (99%), IL-1β (84%), IL-6 (76%) and IL-8 (65%), and at 0.15 μg/mL, it significantly suppressed production of TNF-α and IL-1β by monocytes (77% and 61%, respectively) and of IL-1β and TNF-α by lymphocytes (36% and 16%, respectively). The results obtained in this study show that 1,8-cineole acts as a strong inhibitor of TNF-α and IL-1β and strengths its use as an agent to control airway mucus hypersecretion by cytokine inhibition, suggesting long-term treatment to reduce the severity of asthma, sinusitis and chronic obstructive pulmonary disease. IL-1β, a molecular form of IL-1 (a potent proinflammatory cytokine), is one of the most important mediators of inflammation, which plays important roles in the reactions involved in the acute phase response. It is produced by blood monocytes, macrophages, and dendritic cells, among other cells in the body, and when liberated in small amounts elicits hypotension, fever, and the release of adrenocorticotrophic hormone. It also stimulates the production of cytokines such as IL-6 which, in turn, induces the synthesis of hepatic acute-phase proteins (*i.e.*, serum amyloid A and C-reactive protein) and stimulates the synthesis of adhesion molecules in endothelial cells and leucocytes, producing leukocytosis and thrombocytosis [1].

The efficacy of the acute treatment with 1,8-cineole on reducing inflammatory airway parameters has been assessed in the guinea pig by submitting ovalbumin (OVA)-sensitized animals to antigenic challenge (OVA) with or without pre-treatment with a single dose of inhaled 1,8-cineole. The airway inflammatory parameters were reduced or absent in 1,8-cineole-treated animals, and they included reduced levels of the proinflammatory cytokines TNF-α and IL-1β in bronchoalveolar lavage fluid (BALF), impairment of the OVA-induced increase of MPO activity in BALF, and preventive action in the reduction of the mucociliary clearance induced by the antigen presentation. Additionally, the acute treatment impaired the development of airway hyperresponsiveness to carbachol (a cholinomimetic drug that binds and activates the acetylcholine receptor) in isolated tracheal rings [24].

Further action of 1,8-cineole in the airway was shown in a study carried out in guinea pigs and Wistar rats involving the broncodilatory activity of this monoterpene when compared to the effects of phenoterol, an adrenergic beta-2 agonist that is used as a bronchodilator and tocolytic. 1,8-Cineole (1–30 mg/kg) and phenoterol exhibited similar efficacy in decreasing, *in vivo*, rat bronchial resistance (66.7 ± 3.2% *vs*. 72.1 ± 5.3%, respectively), showing a relaxant activity of 1,8-cineole in the rat and guinea pig (nonsensitized and ovalbumin-sensitized) airway smooth muscle by a nonspecific mechanism [25]. In humans, a double-blind, placebo-controlled multi-center-study was performed employing 242 patients with stable chronic obstructive pulmonary disease that received 200 mg of cineole or placebo three times daily as concomitant therapy for six months during winter-time. The therapy with 1,8-cineole reduced exacerbations as well as dyspnea and improved lung function and health status. The study suggested that 1,8-cineole acted as an active controller of airway inflammation in chronic obstructive pulmonary disease by interfering in the pathophysiology of airway inflammation of the mucus membrane [26].

The assessment of the anti-inflammatory effects of *Artemisia princeps* Pamp essential oil and its main constituents 1,8-cineole and α-terpineol against *Gardnerella vaginalis*-induced vaginosis and vulvovaginal candidiasis in mice revealed that the intravaginal treatment with the essential oil or the monoterpenes produced a significant decrease in the number of viable *G. vaginalis* and *Candida albicans* in the vaginal cavity and MPO activity in vaginal tissues. They also inhibited the expressions of proinflammatory cytokines (IL-1β, IL-6 and TNF-α), cyclooxigenase-2 (COX-2), inducible nitric oxide synthase (iNOS), and the activation of nuclear transcription factor κappa B (NF-κB). In addition, they increased the expression of anti-inflammatory cytokine IL-10, and inhibited the expressions of proinflammatory cytokines and the activation of NF-κB in LPS-stimulated peritoneal macrophages, being α-terpineol the strongest inhibitory effector of the expressions of proinflammatory cytokines and NF-κB activation [27]. NF-κB is a ubiquitous rapid response transcription factor in cells involved in immune and inflammatory reactions. After its activation, NF-κB migrates to the cell nucleus to induce the expression of cytokines, such as TNF-α, IL-1β, IL-6, COX-2 and adhesion molecules (L-selectins, ICAM-1), which are important for the genesis of inflammatory signals [14,28].

In a study developed by Ciftci and collaborators [29], the toxic effects of 2,3,7,8-tetrachlorodibenzo-*p*-dioxin (TCDD—a persistent environmental pollutant) on the percentage of T-cell subsets and B-lymphocyte, and the effectiveness of 1,8-cineole and β-myrcene (myrcene) on this toxicity in rats were examined. Following oral administration of 1,8-cineole (100 mg/kg/day) and myrcene (100 and 200 mg/kg/day) dissolved in corn oil with or without TCDD for 30 and 60 days, the blood samples analysis showed that TCDD significantly reduced the percentage of lymphocyte subsets of CD3+, CD4+, CD161+, CD45RA, CD4+CD25+ and total lymphocyte, but significantly increased the percentage of CD8+ cells. On the other hand, 1,8-cineole and myrcene significantly decreased CD8+ cells levels but increased CD3+, CD4+, CD161+, CD45RA, CD4+CD25+ and total lymphocytes, indicating that these monoterpenes displayed immunomodulatory effects and eliminated TCDD-induced immune suppressive effects in rats.

The anti-inflammatory activity of the monoterpene l-menthol and mint oil was investigated by Juergens and collaborators [15] in human monocytes *in vitro* using LPS-stimulated monocytes followed by the assessment of IL-1β production and the arachidonic acid metabolism by measuring the inflammation mediators LTB4 and PGE2 as indicators for both the lipoxygenase and the cyclooxygenase pathways, respectively. l-Menthol significantly reduced the production of LTB4 (64.4 ± 10%), PGE2 (−56.6 ± 8%), and IL-1β (−64.2 ± 7%), while mint oil exhibited similar effects on LTB4 and IL-1β. The results suggest preferable anti-inflammatory effects of l-menthol in human monocytes, indicating the potential therapeutic efficacy of this monoterpene for treatment of chronic inflammatory disorders such as bronchial asthma, colitis and allergic rhinitis [15]. Furthermore, the anti-allergic effects of peppermint oil, l-menthol, menthone, neomenthol, and 1,8-cineole (chewing gum constituents) were examined in type I allergic reactions, in which l-menthol, menthone and 1,8-cineole suppressed antigen-induced histamine release from rat peritoneal mast cells. In another study, the oral administration of 1,8-cineole and intraperitoneal injection of peppermint oil, l-menthol, menthone and 1,8-cineole inhibited passive cutaneous anaphylaxis (PCA) of guinea pigs as demonstrated by Arakawa and Osawa [30]. The comparison of the clinical efficacy of flavored and non-flavored chewing gums in allergic rhinitis (pollenosis) showed that the peppermint gums enriched with l-menthol, 1,8-cineole, as well as geraniol or citronellol, more effectively attenuated rhinitis symptoms than the non-flavored gum and normal peppermint flavored gum [30,31].

Previously, the comparison between the actions of menthol and retinoic acid (RA) on human leukocyte cell lines showed that RA decreased the number of interleukin-6 receptors (IL-6R) on these cell lines, including the myeloma cell line AF10, and 2 B-cell hybridomas that correspond to cells at earlier stages of B-cell development, while menthol also suppressed the IL-6R expression and inhibited cell growth [32].

### 3.2. Thymoquinone and Thymohydroquinone

Thymoquinone is the main monoterpene of the volatile oil obtained from the seeds of *Nigella sativa* L. (Ranunculaceae), a plant used in folk medicine to treat eczema, asthma, bronchitis and inflammation [33]. Other biological properties of this species include anti-inflammatory, immune stimulation, and respiratory stimulatory effects [34,35,36], among others, all of which have been attributed to the presence of thymoquinone. Several studies have shown the effect of this monoterpene on airway inflammation in mouse (Balb/c) and rat (Sprague-Dawley) models of allergic asthma. The inflammatory reaction in such cases comprises a series of responses that includes increase in the number of T helper type 2 (Th2) cytokine levels (IL-4, IL-5, and IL-13), eosinophils and mast cells, mucus hypersecretion, and production of immunoglobulin E (IgE) [37,38,39,40]. Prostaglandin D2 (PGD2), an important product of arachidonic acid metabolism by cyclooxygenases, is also produced by inflammatory cells in the airways [41], activated Th2 cells and mast cells [42] to the point that the overproduction of PGD2 results in an increased eosinophilic airway inflammation and Th2 cytokine production in allergic mice [43].

In this context, two experiments carried out in Balb/c mice sensitized and challenged through the airways with OVA evidenced that the intraperitoneal injection of thymoquinone (3 mg/kg) exhibited anti-inflammatory activity during the allergic response [37,43]. In the study developed by Mezeyan and collaborators [37], sensitized mice showed a significant increase in PGD2 production in the airways followed by the development of inflammatory responses characterized by increased inflammatory cell numbers and Th2 cytokine levels in the bronchoalveolar lavage fluid, lung airway eosinophilia, goblet cell hyperplasia, and the induction of COX-2 protein expression in the lung, which were all effectively reduced after the administration of thymoquinone.

Similar results were obtained by Gazzar and collaborators [44], in which thymoquinone treatment also produced a marked decrease in lung eosinophilia and production of Th2 cytokines both *in vivo*, in the bronchoalveolar lavage fluid, and *in vitro*, following stimulation of lung cells with OVA. Moreover, thymoquinone decreased the elevated serum levels of OVA-specific IgE and IgG1 and inhibited allergen-induced lung eosinophilic inflammation and mucus-producing goblet cells. Together these findings suggest that thymoquinone exerts anti-inflammatory activity during the allergic response in the lung through the inhibition of eosinophil infiltration, PGD2 synthesis and Th2-driven immune response, giving further support to the role played by the oil of *N. sativa* and thymoquinone in alleviating allergic airway inflammation.

In another experimental procedure, the anti-inflammatory activity of thymoquinone extracted from the oil of *N. sativa* seeds was demonstrated on Sprague-Dawley rats with arthritis induced by Freund’s incomplete adjuvant (immunization with autologous or heterologous type II collagen in adjuvant). Rheumatoid arthritis is a chronic inflammation characterized by symmetrical polyarthritis and systemic involvement in humans. In mouse or rat models, the collagen-induced arthritis causes proliferative synovitis with infiltration of polymorphonuclear and mononuclear cells, pannus formation, cartilage degradation, erosion of bone and joint fibrosis [45]. After induction of arthritis, the evaluation of TNF-α and IL-1β production, since these pro-inflammatory cytokines are found in high levels in the arthritic joints of rodents with collagen-induced arthritis, and the signs of inflammation on the claw as well as the radiological signs, showed that thymoquinone (2.5 and 5 mg/kg, p.o.) significantly reduced clinical and radiological arthritis scores and TNF-α and IL-1β levels, corroborating the anti-inflammatory action of this substance [46].

Epidemiological studies indicate that the offspring of mothers who expressed a diabetic condition during pregnancy are seven times more likely to develop health complications later in life than the offspring of nondiabetic mothers. In this context, an experimental assay was carried out to verify whether supplementation with a natural antioxidant, *i.e.*, thymoquinone, in female rats with streptozotocin (STZ)-induced gestational diabetes (GD) improved diabetic complications and T cell immune responses in their offspring [47]. The supplementation of diabetic mothers with the monoterpene during pregnancy and lactation periods exerted a significant effect on the mean body weight of neonates and significantly increased the IL-2 level and T cell proliferation, restoring both circulating and thymus homing T cells in the offspring. These results suggest that nutritional supplementation of diabetic pregnant mothers with thymoquinone during pregnancy and lactation improves diabetic complications and provides protective effect in later life by maintaining an efficient T cell immune response in the offspring.

The *in vitro* anti-inflammatory activity of compounds derived from *N. sativa* seeds, such as thymohydroquinone, thymol and thymoquinone were investigated using COX-1 and COX-2 assays. They all exhibited general anti-inflammatory activity, particularly thymol that most effectively inhibited COX-1 while thymohydroquinone and thymoquinone were more active on COX-2 [48]. Conversely, Juhás and collaborators [49] investigated the anti-inflammatory activity of thymoquinone and borneol on TNBS-induced colitis in mice and observed a significant decrease in proinflammatory cytokine (IL-1β and IL-6) mRNA expression in colon tissue in the borneol-treated groups of mice, but surprisingly not in the thymoquinone-treated animals.

### 3.3. Borneol, l-Borneol, Bornyl Acetate, and Terpineol

The monoterpene borneol has been shown to be a mast cell membrane stabilizer that can be used as a type I allergy inhibitor. In a rat experimental assay, borneol was found to exert inhibitory effects on the release of histamine from abdominal mast cells by 40.4% [50]. The evaluation of its anti-inflammatory as well as analgesic effects were also performed in the mouse hot-plate test, acetic-acid-induced twisting test, abdominal cavity capillary permeability increase test induced by acetic acid, and the rat pedal swelling test induced by carrageenan [51]. At the same dose level, borneol significantly decreased the foot swelling of rats, increased pain threshold and inhibited twisting response of mice. The results obtained, however, indicated that the analgesic action of borneol was stronger than its anti-inflammatory action.

In another study, the neuroprotective effects of borneol were investigated in an *in vitro* ischemic model of oxygen-glucose deprivation followed by reperfusion (OGD/R) [52]. Borneol was able to reverse OGD/R-induced neuronal injury, nuclear condensation, intracellular reactive oxygen species (ROS) generation, and mitochondrial membrane potential dissipation. It also reduced the nitric oxide (NO), the increase of iNOS enzymatic activity, the upregulation of iNOS expression, and inhibition of caspase-related apoptotic signaling pathway. Furthermore, borneol blocked NF-κB p65 nuclear translocation induced by OGD/R and inhibited the degradation of proinflammatory factor release and nuclear factor of kappa light polypeptide gene enhancer in B-cells inhibitor, alpha (IκBα—a member of a family of cellular proteins that function to inhibit the NF-κB transcription factor). In view of these properties, it is believed that borneol protects against cerebral ischemia/reperfusion injury through multifunctional cytoprotective pathways, indicating that the inhibition of IκBα-NF-κB and translocation signaling pathway might play a significant role in the neuroprotection played by this compound.

Previously, the effects of borneol on inflammation in focal cerebral ischemia-reperfusion rats were also evaluated followed by the detection of changes of neutrophils by immunohistochemestry stain of inter-cellular adhesion molecule-1 (ICAM-1), TNF-α and IL-1β [53]. Rats treated with borneol injection had fewer ICAM-1 positive vessels, IL-1β positive cells, TNF-α positive cells, and number of neutrophils, suggesting that this monoterpene attenuated focal ischemia damage partly through its anti-inflammatory property. Borneol was also reported to display anti-fibrosis effect probably due to its inhibitory action on fibroblasts mitosis, collagen and tissue inhibitors of metalloproteinase 1 (TIMP-1) production as evidenced in an experimental model developed by Jian-Ping and collaborators [54], who attempted to use borneol as a penetration enhancer for a formula used to treat oral submucous fibrosis.

Borneol and terpineol are two monoterpenes extracted from Wu Hu Tang, a Chinese formulation which consists of seven crude drugs that has been used for the treatment of asthma for hundreds of years. The study of their effect on isolated tracheal smooth muscle in guinea pig showed that both compounds prevented histamine-induced *in vitro* bronchoconstriction of guinea pig, thus indicating antiasthmatic effect that could be the base of the antiasthmatic action of Wu Hu decoction [55]. The analysis of the effects of the essential oil obtained from the twigs of *Cinnamomum osmophloeum* Kaneh., and two of its main constituents, namely l-borneol and l-bornyl acetate, on NO and PGE2 production in LPS-activated RAW 264.7 macrophages evidenced that the twig essential oil and the two monoterpenes exhibited excellent anti-inflammatory effects, corroborating their use as a potential source for natural health products [56]. In a previous study, bornyl acetate, the main ingredient of *Amomum villosum* volatile oil, was shown to possess analgesic and anti-inflammatory activities as it was able to decrease the writhing reaction caused by acetic acid and the pain caused by the hot-plate, and to suppress ear swelling caused by dimethylbenzene, giving further support to the anti-inflammatory action exerted by bornyl acetate [16].

The significance of CD4+ and CD8+ T lymphocytes, interferon gamma (IFN-γ)/IL-4, macrophages and IL-10 in the uterus in early embryo loss (or resorption) and anti-abortive effect and immunological modulation of the maternal-fetal interaction with bornyl acetate have been evaluated in several studies [57,58,59]. Bornyl acetate was administered to mice at days 4–7 of gestation and LPS was injected on day 7 to induce abortion. The number of CD4+ T lymphocytes in the uterus of LPS-induced abortion mice was significantly increased, but no significant difference in the number of CD8+ T lymphocytes was observed, although the rate of CD4+/CD8+ was also increased significantly [57]. The mean value of IFN-γ/IL-4 in LPS-treated mice was higher than that of bornyl acetate [58] while the levels of IL-10 were significantly reduced in the uterus with LPS treatment. The amount of macrophages in the uterus of LPS-induced abortion mice was much higher than that of control mice, but with the administration of bornyl acetate combined with another compound (quecertin), used to prevent LPS-induced abortion, the effect on anti-LPS-induced abortion was more significant. The IL-10 content was close to normal, while the amount of macrophages was decreased at a significant level from that of LPS-induced abortion group [59]. It is believed that in the LPS-treated mouse uterus, the increase of CD4+/CD8+ T cells as well as the decrease of IL-10 and the increase of macrophage number could be related with embryo loss, and bornyl acetate could be exerting anti-abortive effect through modulation of maternal-fetal interface immunity balance [59].

### 3.4. Thymol, Carvacrol, Linalool and Linalyl Acetate

Elastase, a proteinase produced by activated human neutrophils, is considered a marker of inflammatory diseases; therefore one strategy to protect tissue against inflammation is to inhibit excessive elastase activity. Thymol, one of the major constituents of thyme oil, seems to interfere with elastase activity as evidenced by the reduced release of this proteinase by human neutrophils stimulated with the synthetic chemotactic peptide *N*-formyl-methionyl-leucyl-phenylalanine (fMLP) and incubated with increasing amounts of thymol (10, 20 μg/mL). It is believed that due to the hydrophobic nature of thymol, this compound can approach and inactivate calcium channels, thus triggering a corresponding reduction in elastase, since the behavior of cytosolic calcium mobilization resembles that of elastase, thereby suggesting that they may be related [60]. The *in vitro* anti-inflammatory activity of compounds derived from *N. sativa* seeds, such as thymol was investigated using COX-1 and COX-2 assays. In this test, thymol effectively inhibited COX-1 [47].

The *in vivo* test on the chorioallantoic membrane of the fertilized hen’s egg (CAM assay) is another experiment model used to determine antiangiogenic, anti-inflammatory and toxic effects of individual compounds or complex plant extracts. An assessment of the effects of the oil obtained from the aerial parts of *Origanum onites* L., a common spice and medicinal plant, and some of its major constituents, including thymol, carvacrol, *p*-cymene, and γ-terpinene, showed that neither the essential oil nor the monoterpenes exhibited a strong anti-inflammatory or antiangiogenic property in the CAM assay at 10–250 μg/pellet [61]. Thymol was also shown to inhibit inducible lymphocyte proliferation in a concentration-dependent manner (62.8% at 50 μg/mL to 89.8% at 200 μg/mL) as evidenced in the investigation of the effects of the organic extracts prepared with plants popularly used to treat infections and inflammatory diseases, namely *Thymus vulgaris*, *T. daenensis* and *Zataria multiflora* (Labiatae), on mitogen (PHA)-stimulated peripheral blood lymphocytes by using a cell proliferation assay [62].

Furthermore, thymol obtained from *Lippia gracilis* Schauer is indicated as a potential agent in the treatment of inflammation and wound healing. The essential oil of this species has been shown to exert antimicrobial activity and in folk medicine it is used externally to treat burns and wounds [63]. In the carrageenan-induced paw edema followed by analysis of MPO activity (used as an indicator of neutrophils), rats treated with thymol (10, 30 and 100 mg/kg, i.p.) exhibited significant reduction of the edema, inhibition of MPO activity and decreased leukocyte influx to the injured area. In mice, the i.p. administration of thymol at the same dose levels produced marked inhibition of peritonitis induced by carrageenan injection into the peritoneal cavity. To test the healing potential of thymol, wounds made on the back of rats were dressed with collagen-based containing thymol films (1.2 mg). After seven and 14 days of treatment, wounds dressed with thymol films exhibited more prominent wound retraction rates, improved granulation reaction, better collagenization density and arrangement during the process of wound healing when compared with wounds treated with collagen-dressed films without thymol [64]. These data provide further support to the pharmacological action displayed by the essential oil of *L. gracilis*, suggesting that thymol plays an important role in these actions.

Carvacrol (2-methyl-5-[1-methylethyl]phenol) is a monoterpene present in the essential oil of various aromatic plants and spices such as *N. sativa* L., *T. vulgaris* L., and *Origanum* species, among others [65,66,67]. These plants are used in folk medicine for treatment of pain, arthritis, asthma and headache [65], and their medicinal properties have been attributed to the presence of high concentrations of active monoterpenes, such as carvacrol, to which a number of significant biological effects, including anti-inflammatory, have been described [68]. For instance, the *in vitro* COX-1 and COX-2 assays showed that carvacrol (0.1, 1, 10, 50 and 100 μM) significantly inhibited production of PGE2 catalysed by COX-2 (IC_50_ value of 0.8 μM) in a similar way as the positive control drugs, indomethacin (COX antagonist) and N-[2-(cyclohexyloxy)-4-nitrophenyl]-methanesulphonamide (NS-398—a selective COX-2 inhibitor) (IC_50_ = 0.7 μM and 0.8 μM, respectively). COX-1 was inhibited approximately at the same rate (IC_50_ of 0.7 μM for carvacrol, and 0.6 μM for indomethacin), suggesting a non-selective inhibition of both enzyme isoforms. In view these findings, it is believed that the anti-inflammatory action of carvacrol is related to its inhibitory effect on PG production mediated via arachidonic acid pathway [69].

In a different research model, Mariko and collaborators [70] also assessed the effect of carvacrol on COX-2 expression through the activity of peroxisome proliferator-activated receptors (PPARs). These receptors are ligand-dependent transcription factors belonging to the nuclear receptor superfamily and also known to modulate COX-2 expression, and vice versa. By use of essential oils derived from various plants, including thyme, clove, rose, eucalyptus, fennel, and bergamot, it was demonstrated that the oils suppressed COX-2 promoter activity in cell-based transfection assays using bovine arterial endothelial cells, and carvacrol, obtained from thyme oil, was appointed as the major suppressor of COX-2 expression and an activator of PPARα and γ. Carvacrol also inhibited LPS-induced COX-2 mRNA in human macrophage-like U937 cells and protein expression, suggesting that this monoterpene regulates COX-2 expression through its agonistic effect on PPAR γ.

The role of carvacrol as anti-inflammatory agent was further investigated by its association with stress proteins, such as the heat-shock protein (Hsp) family. These proteins are up-regulated by cells in inflamed tissue and can be used as “biomarkers” for the immune system to monitor inflammation. The hypothesis that boosting of endogenous Hsp expression can restore effective immunoregulation through T cells specific for stress proteins was tested by evaluating the stress protein expression manipulated *in vivo* and *in vitro* with carvacrol. This monoterpene was shown to possess the ability to coinduce cellular Hsp70 expression *in vitro* and, upon intragastric administration, in Peyer’s patches of mice *in vivo*. It specifically promoted T cell recognition of endogenous Hsp70, as demonstrated *in vitro* by the activation of Hsp70-specific T cell hybridoma and *in vivo* by amplified T cell responses to Hsp70. The administration of carvacrol also increased the number of CD4+CD25+FoxP3+ T cells, systemically in the spleen, and locally in the joint, and almost completely suppressed proteoglycan-induced experimental arthritis. These results indicate that a food component can increase protective T cell responses to a self stress protein and down-regulate inflammatory disease, implying that the immune system can respond to diet [67].

Additionally, the effects of carvacrol were examined in mice fed with a high-fat diet (HFD), an important model of obesity, as well as the potential underlying mechanisms focusing on the gene expression involved in adipogenesis, thermogenesis and inflammation. For this purpose, male C57BL/6N mice received a normal diet or were fed with HFD and with 0.1% carvacrol-supplemented diet (CSD). Mice fed with CSD showed reduced body weight gain, visceral fat-pad weights and plasma lipid levels compared with mice fed with HFD. The dietary carvacrol supplementation significantly reversed HFD-induced up-regulations of adipose tissue genes and protein associated with the signaling cascades that lead to adipogenesis and inflammation. These findings demonstrate that carvacrol prevented obesity in HFD-fed mice, suggesting a possible visceral adipogenesis inhibition by suppressing bone morphogenic protein-, fibroblast growth factor 1- and galanin-mediated signaling. It seems that it also attenuates the production of pro-inflammatory cytokines in visceral adipose tissues by inhibiting toll like receptor 2 (TLR2)- and TLR4-mediated signaling. TLRs are a class of proteins that play a key role in the immune system. When microbes penetrate physical barriers such as the skin or intestinal tract mucosa, they are recognized by TLRs, which activate immune cell responses [71].

The study about the effect of carvacrol on porcine blood lymphocytes revealed that this compound inhibited the proliferation of purified lymphocytes (IC_50_ = 182 ± 67 μM in MTT assays), which was probably caused by apoptotic cell death, as determined by annexin-V binding and caspase-3 activation. However, these effects were not specific for lymphocytes, since carvacrol similarly induced apoptosis and suppressed proliferation in the porcine enterocyte cell line IPEC-1 [72]. The anti-inflammatory effect of carvacrol was also evaluated on the model of carrageenan-induced pleurisy and mouse paw edema, and the LPS-induced nitrite production in murine macrophages. Carvacrol (1, 10, and 100 μg/mL) significantly decreased the LPS-induced nitrite production *in vitro* and did not produce citotoxicity in the murine peritoneal macrophages *in vitro*. In addition, it significantly reduced TNF-α level in pleural lavage and suppressed the recruitment of leukocytes without altering the morphology of these cells [73]. In another study, carvacrol, found in the essential oil of many Chinese medicinal herbs, dose-dependently triggered intracellular calcium mobilization in Jurkat T-cells and THP-1 monocytic cells (HTP-1 = human acute monocytic leukemia ***cell*** line), and stimulated the active phosphorylation of the p38 subgroup of mitogen-activated protein kinases (MAPKs) in both cell types. In contrast, it selectively activated the extracellular-signal-regulated kinase (ERK) subgroup in Jurkat T-cells, and stimulated the JNK subgroup in THP-1 monocytic cells. These findings suggest that the essential oil components of the Chinese herbs such as carvacrol may effectively modulate the functions of immuno-responsive cells via different intracellular signaling pathways [74].

The pharmacological action of carvacrol was further investigated by Silva and collaborators [75] in various models of inflammation and induced gastric ulcers in Wistar rats and Swiss mice. The treatment with carvacrol significantly reduced paw edema induced by histamine, dextran and substance P by 46%, 35% (50 mg/kg) and 46% (100 mg/kg), respectively. It also significantly decreased ear edema induced by 12-*O*-tetradecanoylphorbol-13-acetate (TPA) and arachidonic acid by 43% and 33%, respectively, while indomethacin (positive control substance) (0.5 mg per ear) showed a more effective inhibition of edema formation in both tests (56% and 57%, respectively). In the acetic acid-induced gastric lesion test, the oral treatment with carvacrol (25, 50 and 100 mg/kg) for 14 days improved the healing process of gastric ulcers and significantly reduced the area of lesion by 60%, 91% and 81%, respectively. The experimental protocols employed in this study suggest that carvacrol acts on different pharmacological targets through distinct mechanisms, interfering with the production of inflammatory mediators favoring its anti-inflammatory and healing effects.

Linalool and linalyl acetate are monoterpenes reported to be major volatile components of the essential oils of several aromatic species. An assessment of the effects of (−)-linalool on chronic inflammatory hypersensitivity induced by intraplantar injection of complete Freund’s adjuvant (CFA) was carried out on adult female Swiss mice treated with a single intraperitoneal (i.p.) injection of (−)-linalool (50 or 200 mg/kg) or multiple treatments given chronically (twice daily for 10 days; 50 mg/kg, i.p.). In both treatment protocols, (−)-linalool significantly reduced CFA-induced mechanical hypersensitivity and produced effective reduction in CFA-induced paw edema following the acute treatment [76].

A previous study showed the anti-inflammatory properties of (–)-linalool, its racemate form, *i.e.*, (±)-linalool, and linalyl acetate (a monoterpene ester) by using carrageenan-induced edema in male Wistar rats [77]. The systemic administration (abdominal subcutaneous injection) of (–)-linalool and (±)-linalool effectively reduced paw edema. At a dose level of 25 mg/kg, (–)-linalool produced a delayed and more prolonged effect, while (±)-linalool induced a significant reduction of the edema only one hour after carrageenan administration. The treatment with higher doses (50 and 75 mg/kg) of (–)-linalool produced the maximum inhibitory effect against edema one hour after carrageenan injection (58% and 60%, respectively), whereas (±)-linalool (50 and 75 mg/kg) did not exert any anti-inflammatory activity one hour after carrageenan induction, but induced a significant effect after three (51% and 38%, respectively) and five hours (45% and 34%, respectively). In contrast, linalyl acetate was less effective and more delayed than (–)-linalool and (±)-linalool to produce inhibition of the inflammatory process in the rat’s paw.

### 3.5. α-Terpinene, α-Terpineol, Terpinen-4-ol, α-Carveol, Menthone, Pulegone, Geraniol, Citral, Citronellol, Perillyl Alcohol, Perillic Acid, β-Myrcene, Carvone and Limonene

The anti-inflammatory activity of other monoterpenoids was reported in experimental protocols which showed selective inhibition of ovine COX-2 activity by α-terpinene, α-terpineol, α-carveol, menthone and pulegone. α-terpineol, for instance, showed higher COX-2 activity inhibition than aspirin, the most popular NSAID [78]. The investigation of topical anti-inflammatory effect of some components of the essential oil of *Zingiber cassumunar* showed that terpinene-4-ol and α-terpinene, but not γ-terpinene, effectively inhibited edema formation, the latter being the most active constituent and twice as potent as the reference drug diclofenac, a NSAID, taken to reduce inflammation and as analgesic in certain conditions [79]. With *Melaleuca alternifolia*, a native Australian tea tree, the anti-inflammatory activity of the essential oil obtained from this plant and three of its major constituents, *i.e.*, terpinen-4-ol (42%), α-terpineol (3%) and 1,8-cineole (2%), was assessed by its ability to reduce the production *in vitro* of TNF-α, IL-1β, IL-8, IL-10 and PGE2 by LPS-activated human peripheral blood monocytes. The tea tree oil was toxic for monocytes at a concentration of 0.016% v/v, whereas terpinen-4-ol significantly suppressed LPS-induced production of TNFα, IL-1β and IL-10 by approximately 50% and PGE2 by approximately 30%, after 40 h. The individual testing of these monoterpenes showed that only terpinen-4-ol suppressed the production after 40 h of TNFα, IL-1β, IL-8, IL-10 and PGE2 by LPS-activated monocytes [80].

Geraniol is a monoterpene present in the essential oils of fruits and herbs and has been reported to act in cancer chemoprevention. The immunosuppressive activity of geraniol was studied *in vitro* with lymphocyte proliferation assays and *in vivo* in a rat cardiac allograft transplant model (MHC disparate Wistar-Furth to Lewis rat heterotopic abdominal heart transplant model). In the proliferation assay, there was a concentration-dependent inhibition of lymphocyte proliferation, while in the cardiac allograft transplant model, geraniol significantly prolonged graft survival. The results indicated that geraniol had a modest *in vitro* and *in vivo* immunosuppressive activity, however when combined with cyclosporine A (CsA), an immunosuppressant drug used in post-allogeneic organ transplant to reduce the activity of the immune system, there was an additive immunosuppressive activity that could allow a dose reduction of CsA [81].

Mevalonate kinase deficiency (MKD) is a rare disorder characterized by recurrent inflammatory episodes. Defective synthesis of isoprenoids and drugs such as aminobisphosphonates, which are known to inhibit the mevalonate pathway causing a relative defect in isoprenoids synthesis, have been associated with the inflammatory phenotype in MKD patients. Therefore, the inhibition of the mevalonate pathway through genetic defects or aminobisphosphonates reduces the production of intermediate compounds, in particular geranylgeranyl-pyrophosphate (GGPP), which is associated with the consequent increased IL-1β release in monocytes. Geraniol is an example of isoprenoid that enters the mevalonate pathway and is capable of reverting the genetic or pharmacological inhibition. In this context, the effect of geraniol on bacterial induced-inflammation was tested in a monocytic cell line (Raw 264.7) and in Balb/c mice treated with pamidronate, a nitrogen containing biphosphonate used to prevent osteoporosis. Geraniol reduced the levels of inflammatory markers induced by pamidronate stimuli *in vitro* and *in vivo*, suggesting that this monoterpene could be used as a novel alternative therapeutic approach for MKD, and also be considered for the evaluation of possible inflammatory side-effects of aminobisphosphonates [82]. An earlier study using a mouse model for typical MKD inflammatory episode showed that after treatment of BALB/c mice with aminobisphosphonate alendronate and bacterial muramyldipeptide, the addition of exogenous geraniol and geranylgeraniol effectively prevented inflammation induced by alendronate-muramyldipeptide, giving further evidence of a possible role for these monoterpenes in the treatment of MKD in humans [83].

Geranium oil is popularly used to treat diarrhea, dermatitis, and intestinal inflammation in East Asia. The investigation of the anti-inflammatory effects of geranium oil and two of its constituents, geraniol and citronellol, on LPS-induced NO and PGE2 production in RAW 264.7 macrophages showed that geraniol and citronellol inhibited NO and PGE2 production in a dose-dependent manner. The inhibitory action of geraniol occurred concomitantly with the decrease in protein and mRNA expression levels of iNOS, whereas citronellol inhibited only iNOS enzymic activity. The addition of geraniol and citronellol reduced the LPS-induced COX-2 protein and mRNA expression levels, but cytosolic degradation of IκBα and upregulation of NF-κB p65 in the nucleus were reversed [84]. Furthermore, geraniol and citronellal obtained from rose oil activated PPARα and γ, and suppressed LPS-induced COX-2 expression in cell culture assays [85]. These findings support the popular use of geraniol and citronellol as anti-inflammatory agents, thus suggesting a therapeutic potential for inflammation-associated disorders [84].

In a different approach, the efficacy of geranium oil and geraniol was tested against vaginal candidiasis as well as their effect on vaginal inflammation and *Candida* growth form by triggering an intravaginal infection of *C. albicans* to estradiol-treated mice. The vaginal application of geranium oil or geraniol successfully suppressed *Candida* cell growth in the vagina and its local inflammation, when combined with vaginal washing, indicating a protective role of the essential oil and its main monoterpene against vaginal candidiasis in mice [86].

The assessment of the effects of essential oils obtained from lemongrass, geranium and spearmint oils and some of their major constituents (geraniol, citronellol, citral and carvone) on neutrophil activation by measuring TNF-α-induced adherence reaction of human peripheral neutrophils showed that all essential oils tested (0.1% concentration) and the major constituents suppressed TNF-α-induced neutrophil adherence. In contrast, very popular essential oils, such as tea tree oil and lavender oil, failed to display any inhibitory activity [87]. Moreover, butylidene phthalide or the monoterpenes geraniol and citronellol, used alone or in combination, was reported to reduce or relieve the syndromes of the inflammation. The suppression of NO synthesis by these compounds was investigated in murine macrophage/monocyte RAW 264.7 cells stimulated with lipopolysaccharides (100 ng/mL) in the presence of different concentrations of geraniol, citronellol or butylidene phthalide. The IC_50_ of geraniol, citronellol or butylidene phthalide was 402.8 μM, 295 μM, and 406.5 μM, respectively, indicating that citronellol more effectively suppressed NO synthesis when compared to geraniol and butylidene phthalide [88].

Furthermore, citronellol has been reported to possess anticancer, anti-inflammatory and wound healing properties. A double-blind, placebo-controlled study investigated whether a traditional Chinese herb complex consisting of a mixture of citronellol and extracts of *Ganoderma lucidum*, *Codonopsis pilosula* and *Angelicae sinensis*, species with proven immunomodulatory functions, improved the immune cell counts of cancer patients (n = 105) receiving chemotherapy and/or radiotherapy. The herb complex significantly decreased leukocyte depletion (14.2% *vs*. 28.2%) and neutrophils (11.0% *vs*. 29.1%), suggesting an improvement in the immune function of patients receiving chemotherapy and/or radiotherapy by increasing their ability to fight off the cancer, and secondary infections that could compromise the treatment and their health [89].

Geraniol and perillyl alcohol were also tested as a possible new therapy for treatment of pancreatic cancer, since previous testing revealed that these monoterpenes exhibited chemotherapeutic potential in pancreatic and other tumor types. When given in combination, these compounds displayed an additive antiproliferative effect against MIA PaCa-2 human pancreatic cancer cells, and when given individually, they all induced a G0/G1 cell cycle arrest that coincided with an increase in the expression of the cyclin kinase inhibitor proteins p21Cip1 and p27Kip1, and a decrease in cyclin A, cyclin B1, and cyclin-dependent kinase (Cdk) 2 protein levels. All these data indicate that geraniol and perillyl alcohol exhibit chemotherapeutic potential by using a p21Cip1- and p27Kip1-dependent antiproliferative mechanism in human pancreatic adenocarcinoma cells [90]. The chemopreventive effect of topical application of perillyl alcohol on 9,10-dimethylbenz(a)anthracene (DMBA)-initiated and TPA-promoted skin tumorigenesis in Swiss albino mice was evidenced after pretreatment with perillyl alcohol (6 and 12 mg/kg body weight) on TPA (2 μg/200 μL of acetone)-induced skin edema, hyperplasia, peroxidase damage and modulation in the activities of various enzymes (catalase, glutathione reductase, glutathione peroxidase, glutathione-S-transferase), and reduced glutathione contents. Perillyl alcohol significantly inhibited ornithine decarboxylase (ODC) activity and [(3)H] thymidine incorporation into epidermal DNA. In promotion phase, a significant decrease in tumor incidence and tumor burden was observed in mice pretreated with perillyl alcohol (12 mg/kg body weight), in addition to inhibition of the Ras/Raf/ERK pathway, and induction of apoptosis in mice skin. All these findings indicate that perillyl alcohol exhibit chemopreventive properties related to the inhibition of oxidative stress responses, inhibition of the Ras cell proliferation pathway and induction of apoptosis in murine skin tumor promotion phase [91]. Other studies also provided further evidence of perillyl alcohol anticancer and chemopreventive properties in rodent models. For instance, perillyl alcohol effectively inhibited human T cell proliferation *in vitro* and prevented acute and chronic rejection in a rat cardiac transplant model. The investigation of the effects of perillyl alcohol on T lymphocytes at the single-cell level showed that this monoterpene disrupted the polarized shape and motility of antigen-specific murine 1E5 T cells and preferentially induced apoptosis in PHA-activated human T cells as well as in 1E5 T cells [92].

The inflammatory process is an important etiological factor that may exert a key role in the development of ethanol induced liver injury; moreover the release of TNF-α and activation of NFκ-B may strongly intensify this process and cause cell damage. The administration of ethanol to rats has been shown to produce a significant increase in various enzymes, such as serum aspartate aminotransferase, alanine aminotransferase, lactate dehydrogenase and hepatic malondialdehyde. It also decreased hepatic reduced glutathione content and activity of various antioxidant enzymes, and increased TNF-α production and NFκ-B activation. The assessment of the protective effects of perillyl alcohol on ethanol-induced acute liver injury in Wistar rats showed that the pretreatment with the monoterpene significantly improved ethanol induced acute liver injury possibly by inhibition of lipid peroxidation, replenishment of endogenous enzymic and non-enzymic defense system, and down-regulation of TNF-α and NFκ-B [93].

Lemongrass (*Cymbopogon citratus* L.) is a popular herb used as a food flavoring, and as analgesic and anti-inflammatory agent. Katsukawa and collaborators [94] reported that the essential oil of lemongrass suppressed COX-2 promoter activity and identified citral as the major component responsible for suppressing COX-2 expression and for activating PPARα and γ. In human macrophage-like U937 cells, citral inhibited LPS-induced COX-2 mRNA and protein expression, and induced the mRNA expression of the PPARα-responsive carnitine palmitoyltransferase 1 gene and the PPARγ-responsive fatty acid binding protein 4 gene, providing important evidence for understanding the anti-inflammatory properties of lemongrass. Further evidence of this monoterpene anti-inflammatory activity was provided in a laboratory-based research that tested citral obtained from *Lippia alba* and *C. citratus* by inducing peritonitis and paw edema with carrageenan in rodents. The pretreatment with citral (100 and 200 mg/kg) significantly decreased the paw edema and the leukocyte migration in the carrageenan-induced migration to the peritoneal cavity [95]. Moreover, citral (3–12 μg/mL) was shown to significantly suppress LPS-induced NO production in a concentration-dependent manner (IC_50_ = 6.5 μg/mL) in RAW 264.7 cells. It also inhibited the transcriptional activity and expression of iNOS, and suppressed the DNA binding activity and nuclear translocation of NF-κB as well as IκB phosphorylation in a concentration dependent manner, suggesting that citral mechanism of action involved the inhibition of NO production through the suppression of NF-κB activation [96].

Limonene, a monoterpene commonly found in species of *Citrus*, and its metabolites have been shown to exhibit chemopreventive and chemotherapeutic properties against different tumours in animal models and clinical trials. In [97], Yoon and collaborators carried out a study to verify the pharmacological and biological effects of limonene on the production of pro-inflammatory cytokines and inflammatory mediators in RAW 264.7 macrophages. Limonene effectively inhibited LPS-induced NO and PGE2 production that included dose-dependent decreases in the expression of iNOS and COX-2 proteins. The evaluation of the inhibitory effects of limonene on other cytokines by measuring TNF-α, IL-1β, and IL-6 levels in the cell supernatants of LPS-stimulated RAW 264.7 macrophages by enzyme-linked immunosorbent assay showed that limonene decreased their expression in a dose-dependent manner. Further information about limonene properties was provided by a study that tested the *in vitro* anti-inflammatory effects of limonene obtained from the peel of a traditional Japanese medicine named yuzu (*Citrus junos* Tanaka) on human eosinophilic leukemia HL-60 clone 15 cells by measuring the level of ROS, monocyte chemoattractant protein-1 (MCP-1), NF-κB, and p38 mitogen-activated protein kinase (MAPK). At a low concentration, limonene (7.34 mmol/L) suppressed the production of ROS for eotaxin-stimulated HL-60 clone 15 cells, while at a higher concentration, limonene (14.68 mmol/L) suppressed cell chemotaxis in a p38 MAPK and decreased MCP-1 production via NF-κB activation comparable to the addition of the proteasomal inhibitor MG132. All these findings suggest that limonene could be used as a potential anti-inflammatory agent for the treatment of bronchial asthma by suppressing cytokines, ROS production, and inactivating eosinophil migration [98].

In the study of ozone as an important inflammatory mediator, Keinan and collaborators [99] hypothesized that the pulmonary inflammation in asthma involves a vicious circle of ozone production and recruitment of white blood cells, which produce more ozone. They also predicted that electron-rich olefins, which are known ozone scavengers, could be used for prophylactic treatment of asthma. Thus, it was postulated that unsaturated monoterpenes could saturate the pulmonary membranes and thereby provide the airways with local chemical protection against either exogenous or endogenous ozone. An examination of the pulmonary function of sensitized rats that inhaled either limonene (unsaturated, ozone scavenger) or 1,8-cineole (saturated, inert to ozone) was carried out and showed that limonene inhalation, but not 1,8-cineole, significantly prevented bronchial obstruction. The anti-inflammatory effect of limonene was also observed in pathological parameters, which exhibited reduced peribronchiolar and perivascular inflammatory infiltrates.

The reduction of tumor cell proliferation and antimutagenicity of the essential oil from *Agastache rugosa* O. Kuntze and limonene, one of its major components, were examined by using four human carcinoma cell lines. The oil exhibited relatively strong antimutagenicity when screened against a genetically engineered Chinese hamster ovary (CHO AS-52) cell line, and displayed a better inhibition effect on cancer cells than limonene. The oil enhanced up to about 200% the growth of B and T cell lines, whereas limonene failed to improve the human T cells. These results indicate that the oil could selectively inhibit the proliferation of human cancer cells better than the individual oil components, possibly involving the synergistic effect of the oil’s major components and/or the combined effects by other unknown minor components in the oil [100].

The effects of limonene on the survival of lymphoma-bearing mice fed with a diet with this monoterpene were demonstrated by Del Toro-Arreola and collaborators [101], who investigated the cell immune response in BALB/c mice sensitized and challenged with 2,4-dinitrofluorobenzene (DNFB). The T-cell subpopulations were measured by flow cytometry and to further investigate the role of limonene and its metabolites, macrophage NO production and lymphocyte proliferation studies were carried out *in vitro* with D-limonene, perillic acid, and perillyl alcohol. Phagocytosis, microbicidal activity, and chemotactic function in peritoneal macrophages were also analyzed. Limonene increased the survival of lymphoma-bearing mice, delayed hypersensitivity reaction to DNFB, phagocytosis and microbicidal activity, and increased NO production in peritoneal macrophages obtained from tumor-bearing mice, indicating a modulatory effect of limonene on the immune response with significant potential for clinical application.

The essential oils from *Conyza bonariensis* and *Porophyllum ruderale* (Asteraceae) were examined for anti-inflammatory activity in the mouse model of pleurisy induced by zymosan (500 μg/cavity) and LPS (250 ng/cavity). Limonene from *C. bonariensis* and β-myrcene from *P. ruderale* were also tested in the LPS-induced pleurisy model and assayed for immunoregulatory activity by measurement of NO inhibition and production of IFN-γ and IL-4. The oral administration of both oils suppressed the LPS-induced inflammation and cell migration in a similar way as that displayed by limonene. β-Myrcene and limonene effectively suppressed NO production as well as the production of IFN-γ and IL-4 at doses below the cytotoxicity of these monoterpenes [102].

The immunomodulatory effect of orange juice and some of its major constituents, namely limonene, linalool and α-terpineol, was shown *in vitro* and *ex vivo* in epithelial buccal cells (KB) through the analysis of the intracellular formation of pro-inflammatory cytokine IL-6 and anti-inflammatory cytokine IL-10 by flow cytometry. Anti-inflammatory effects were displayed when exposure of KB cells to orange juice or its constituents resulted in the suppression of IL-6 formation and, concomitantly, an increase in IL-10 formation. These *in vitro* effects were further analyzed by *ex vivo* experiments, in which exposure of whole blood showed anti-inflammatory action in human macrophages after incubation with α-terpineol [103]. Furthermore, Held and Somoza [104] tested orange juice as well as limonene, linalool and α-terpineol on the intracellular formation of anti-inflammatory cytokine IL-4. Reduced formation of these cytokines was observed after exposure to whole orange juice, while linalool and limonene had no significant action, and α-terpineol exhibited a stimulating activity, showing for the first time the anti-inflammatory effect displayed by α-terpineol on cytokine production in buccal cells.

The assessment of the immunomodulatory activity of limonene, carvone and perillic acid in Balb/c mice showed an increase in the total white blood cells count, the total antibody, and the antibody producing cells in spleen and bone marrow cellularity, suggesting a potentiating effect of limonene, carvone and perillic acid on the immune system [105]. Another study showed that *Minthostachys verticillata* (Griseb) essential oil displayed immunomodulatory effects on cells from allergic patients. Three of its main components, namely pulegone (63.4%), menthone (15.9%), and limonene (2.1%), were tested *in vitro* and *in vivo* for modulatory activity in the immediate-type allergic reaction by measuring IL-13 levels from lymphocytes cultures stimulated with allergen alone or combined with these monoterpenes. Pulegone, menthone and limonene increased cell proliferation, but decreased IL-13 levels, being limonene and the mixture of the three compounds the most active combination. In addition, the essential oil and limonene suppressed mast cell activation and degranulation in the skin when testing passive cutaneous anaphylaxis, being limonene once again the most active compound. They also reduced, in whichever combination, β-hexosaminidase release from basophil with values even lower than those of the antiallergic drug desloratadine. These findings indicate that limonene was the most potent agent displaying immunomodulatory activity, providing a promising natural alternative for the treatment of allergic diseases [106].

### 3.6. Hydroxydihydrocarvone, Fenchone, α-Pinene, (S)-cis-Verbenol, and Piperitenone Oxide

Hydroxydihydrocarvone (HC), a monoterpene obtained by hydration of the natural compound (*R*)-(−)-carvone, was shown to exert anti-inflammatory activity in rodents by use of the carrageenan-induced paw edema and the MPO activity or peritonitis models. The oral administration of HC effectively reduced rat paw edema at 100 and 200 mg/kg, MPO activity at 200 mg/kg, and carrageenan-induced neutrophil recruitment to peritoneal cavity [107]. It is believed that various mechanisms may play a role in the anti-inflammatory effects of HC, since terpenoids are known to display inhibitory activity on the inflammatory signaling cascade of NF-κB by different interactions in this pathway [108], which could produce decreased expression of COX-2, iNOS, and inflammatory cytokines, along with other effects induced by the translocation of NF-κB to its DNA binding site [109].

The anti-inflammatory property of other naturally occurring monoterpenes, such as fenchone, α-pinene, (*S*)-*cis*-verbenol, and piperitenone oxide have also been investigated. Ozbek [110] showed that fenchone (0.05, 0.10 and 0.20 mL/kg) exerted anti-inflammatory action in rats by reducing inflammation by 45.87%, 53.15% and 70.60%, respectively, as opposed to 95.70% reduction with the positive control drug indomethacin in the carrageenan- induced right hind-paw edema model. The effects of *Ugni myricoides* (Kunth) O. Berg essential oil and its major constituent, α-pinene (52.1%), were analyzed in inflammatory and neuropathic models of hypernociception in mice and compared with those of indomethacin or gabapentin, drugs used clinically to treat inflammatory and neuropathic processes. Similarly to indomethacin (5 or 10 mg/kg, p.o.), the oil (5–50 mg/kg, p.o.) significantly prevented mechanical hypernociception induced by carrageenan or complete Freund’s adjuvant (CFA) in mice. In addition, the treatment with the oil (5–25 mg/kg, p.o.), α-pinene (5–50 mg/kg, p.o.), or gabapentin (70 mg/kg, p.o.) also abolished mechanical sensitization induced by CFA, indicating that the effects displayed by *U. myricoides* essencial oil are related, at least in part, to the presence of α-pinene, which shows a potential role for the management of inflammatory and neuropathic pain [111].

(*S*)-*cis*-Verbenol, a natural metabolite from (−)-α-pinene of host pine tree, has been shown to have anti-ischemic activity and to reduce cerebral ischemic injury caused by 1.5-hour middle cerebral artery occlusion followed by 24-hour reperfusion. It significantly prevented neuronal cell death caused by oxygen-glucose deprivation (OGD, 1 h) and subsequent re-oxygenation (5 h and diminished the intracellular level of ROS elevated by OGD/re-oxygenation), and decreased the expression levels of pro-inflammatory cytokines in ischemic brain and immunostimulated glial cells. These findings indicate that (S)-*cis*-verbenol could become a useful therapeutic agent due to its anti-oxidative and anti-inflammatory activities [112].

The essential oil of *Mentha × villosa* Huds (Labiatae) exerts various pharmacological activities, such as antispasmodic effects. A study designed to investigate the antinociceptive action of *Mentha × villosa* oil and one of its main constituents, namely piperitenone oxide, showed that they significantly decreased the writhings induced by acetic acid and the paw licking behavior only during the second phase of the formalin test, an effect that was not reversed by naloxone. However, when evaluated by the hot-plate and tail immersion tests, the oil and the monoterpene exhibited no analgesic activity. These findings demonstrate that *Mentha × villosa* oil and piperitenone oxide have antinociceptive activity and suggest that this effect is probably an indirect anti-inflammatory action, which does not involve the central nervous system [113].

## 4. Conclusions

This review showed the anti-inflammatory profile of monoterpenes that were bioactive on experimental models related to inflammation. The data reported suggests the therapeutic potential of this chemical class as source for the development of novel anti-inflammatory agents. Whereas the bioactive monoterpenes discussed in this review are found in many aromatic and medicinal plants, they should contribute to the anti-inflammatory activity of these species [114]. Therefore, these substances should attract the interest of researchers and pharmaceutical companies for clinical studies and other applications in the therapy of diseases related to inflammatory processes.

## Supplementary Materials

Supplementary materials can be accessed at: http://www.mdpi.com/1420-3049/18/1/1227/s1.
