# Supplementary material for: A Review on Anti-Inflammatory Activity of Monoterpenes
Source: Molecules. 2013 Jan 18;18(1):1227–54. doi: 10.3390/molecules18011227 (PMC6269770; doi:10.3390/molecules18011227)
Supplement: Supplementary file 1 [file molecules-18-01227-s001.pdf]

Table S1. Monoterpenes chemical structures with anti-inflammatory activity.

	Experimental protocol	Anti-inflammatory activity and/or mechanism	Animal tested	Reference
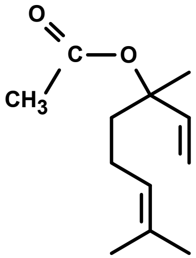 **Linalyl acetate**	In vitro assay with blood mononuclear cells	Immunomodulation<br>NKCA lymphocyte activation (CD69 expression)	Human	[6]
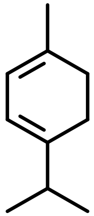 **α-Terpinene**	Popliteal lymph node assay	Immunostimulatory activity	Rat	[7]
	COX-2 assay	Inhibition of COX-2 activity	Ovine	[79]
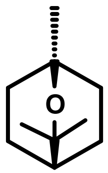 **1,8-Cineole**	Carrageenan-induced paw edema; Cotton pellet-induced granuloma	Reduced paw edema;<br>Reduced granuloma tissue weight	Wistar rat	[13]
	TNBS-induced colitis	Reduced MPO activity	Wistar rat	[17,19,20]
	Bronchial asthma patients	Mucolytic and steroid-saving effect	Human	[21]
	IL-1β and LPS-stimulated mediator production	Reduced TNF-α, IL-1β, LTB4, tromboxane B2 and PGE2 production	Human	[22]

Table S1. *Cont.*

	**Experimental protocol**	**Anti-inflammatory activity and/or mechanism**	**Animal tested**	**Reference**
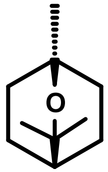 **1,8-Cineole**	Lymphocytes;<br>LPS-stimulated monocytes	Reduced TNF-α, IL-1β, IL-4, IL-5, IL-6 and IL-8 production	Human	[23]
	OVA-sensitization	Reduced airway inflammation;<br>Reduced TNF-α and IL-1β production; Reduced MPO activity in BALF	Guinea pig	[24]
	Gardnerella vaginalis—induced vaginasis;<br>Vulvovaginal candidiasis	Reduced number of *G. vaginalis* and *Candida albicans*;<br>Reduced MPO activity;<br>Reduced TNF-α, IL-1β, IL-6, COX-2, iNOS and NF-kB activation;<br>Increased IL-10 expression	Mice	[27]
	TCDD-induced toxicity	Elimination of TCDD-induced immune suppressive effects;<br>Reduced CD8+;<br>Increased CD3+, CD4+, CD161+, CD4+CD25+ and total lymphocytes	Rat	[29]
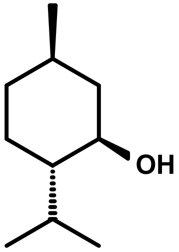 **l-Menthol**	LPS-stimulated monocytes	Reduced IL-1β, LTB4, and PGE2 production	Human	[15]
	PCA-model	Anti-allergic rhinitis	Guinea pig	[30]
	Type I allegic reaction	Reduced histamine release	Rat mast cell	[31]

Table S1. *Cont.*

	**Experimental protocol**	**Anti-inflammatory activity and/or mechanism**	**Animal tested**	**Reference**
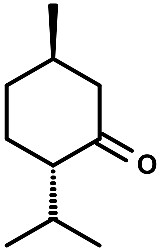 **Menthone**	PCA-model	Anti-allergic rhinitis	Guinea pig	[30]
	Type I allegic reaction	Reduced histamine release	Rat mast cell	[31]
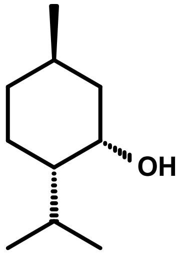 **Neomenthol**	Type I allegic reaction	Reduced histamine release	Rat mast cell	[31]
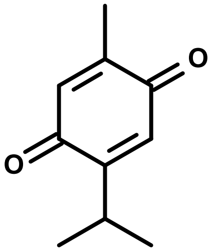 **Thymoquinone**	OVA-sensitization	Reduced production of PGD2, COX-2, IL-4, IL-5, IL-13, IgE and IgG1	Balb/c mice	[37,43]
	Freund`s incomplete adjuvant-induced arthritis	Reduced TNF-α and IL-1β production	Sprague-Dawly rat	[46]
	STZ-induced gestational diabetes	Increased IL-2 production; Increased number of T cells (offspring)	Wistar Rat	[47]

Table S1. *Cont.*

	**Experimental protocol**	**Anti-inflammatory activity and/or mechanism**	**Animal tested**	**Reference**
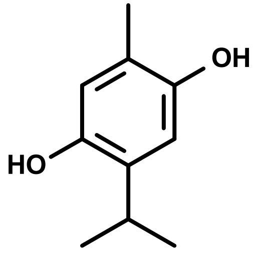 **Thymohydroquinone**	COX-1 and COX-2 assays	Modulatory response; Reduced COX-1 and COX-2 expression		[48]
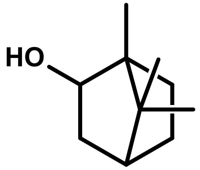 **Borneol**	Type I allergic reaction	Reduced histamine release	Rat mast cell	[50]
	Focal cerebral ischemia reperfusion	Reduced ICAM-I, TNF-α and IL-1β production; Reduced number of neutrophils	Rat	[53]
	TNBA-indiced colitis	Reduced IL-6 and IL-1β mRNA expression	Mice	[49]
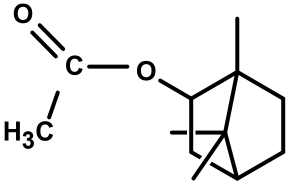 **Bornyl acetate**	LPS-induced abortion	Anti-abortive effect; Reduced number of CD4 and T lymphocytes, macrophages; Reduced rate of CD4+/CD8+; Increased IL-10 production; Reduced IFNγ and IL-4 production	Mice	[57–59]

Table S1. *Cont.*

	Experimental protocol	Anti-inflammatory activity and/or mechanism	Animal tested	Reference
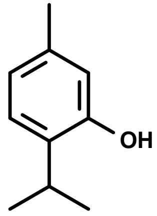 **Thymol**	fMLP-stimulated neutrophils	Reduced release of elastase;<br>Calcium channel inactivation	Human	[60]
	Carrageenan-induced paw edema; Carrageenan-induced peritonitis	Reduced paw edema;<br>Reduced MPO activity;<br>Reduced leukocyte influx	Rat and mice	[63]
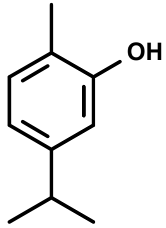 **Carvacrol**	Cell-based transfection assays	Suppression of Cox-2 expression; Activation of PPARα and γ	Bovine arterial endothelial cells	[70]
	LPS-induced COX-2 expression	Inhibition of COX-2 mRNA	Human macrophage-like U937 cells	[70]
	Stress protein expression (T cell specific)	Immunoregulation;<br>Amplified T cell response to Hsp 70;<br>Promotion of T cell recognition of Hsp 70;<br>Increased number of CD4+CD25+FoxP3+ T cells	Mice:<br>Peyer's patches;<br>Hsp70-specific T cell hybridoma	[67]

Table S1. *Cont.*

	Experimental protocol	Anti-inflammatory activity and/or mechanism	Animal tested	Reference
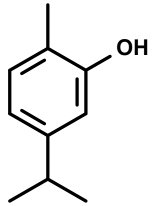 **Carvacrol**	High-fat diet	Attenuation of pro-inflammatory cytokines; Inhibition of TLR2 and TLR4-mediated signaling	C57BL/6N mice (visceral adipose tissue)	[71]
	Blood lymphocytes	Inhibition of lymphocyte proliferation;<br>Annexin-V binding and caspase-3 activation	Porcine	[72]
	Carrageenan-induced pleurisy and paw edema;<br>LPS-induced nitric production	Suppressed recruitment of leukocytes;<br>Reduced TNF-α level and nitrite production	Mice;<br>Murine macrophages	[73]
	Histamine, dextran and substance P-induced paw edema;<br>TPA and arachidonic acid-induced ear edema;<br>Acetic acid-induced gastric lesion	Reduced paw and ear edema;<br>Reduced gastric lesion	Rat and mice	[75]

Table S1. *Cont.*

	**Experimental protocol**	**Anti-inflammatory activity and/or mechanism**	**Animal tested**	**Reference**
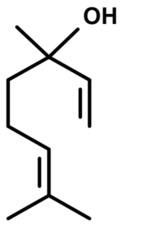 **Linalool**	CFA-induced edema	Reduced mechanical hypersensitivity and paw edema	Mice	[76]
	Carrageenan-induced paw edema	Reduced paw edema	Wistar rat	[77]
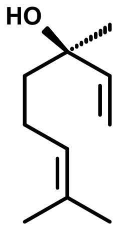 **(-)-Linalool**	Carrageenan-induced paw edema	Reduced paw edema	Wistar rat	[77]
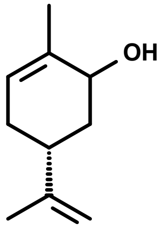 **Carveol**	COX-2 assay	Inhibition of COX-2 activity	Ovine	[78]

Table S1. *Cont.*

	**Experimental protocol**	**Anti-inflammatory activity and/or mechanism**	**Animal tested**	**Reference**
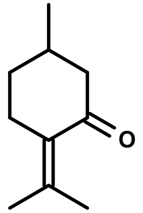 **Pulegone**	COX-2 assay	Inhibition of COX-2 activity	Ovine	[78]
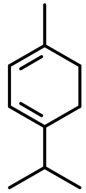 **α-Terpinene**	COX-2 assay	Inhibition of COX-2 activity	Ovine	[78]
	Induced ear edema	Inhibited edema formation	Rats	[79]
	COX-2 assay	Inhibition of COX-2 activity	Ovine	[78]
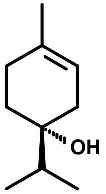 **Terpinene-4-ol**	LPS-activated peripheral blood monocytes	Reduced TNF-α, IL-1β, IL-8 and PGE2 production	Human	[80]
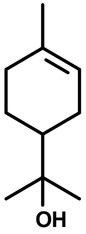 **α-Terpineol**	*Gardnerella vaginalis*- induced vaginasis; Vulvovaginal candidiasis	Reduced number of *G. vaginalis* and *Candida albicans*; Reduced MPO activity; Reduced TNF-α, IL-1β, IL-6, COX-2, iNOS and NF-kB activation; Increased IL-10 expression	Mice	[27]
	COX-2 assay	Inhibition of COX-2 activity	Ovine	[78[
	Epithelial buccal cells	Suppressed IL-6 production; Increased IL-10 production	Human; macrophage	[103]

Table S1. *Cont.*

	**Experimental protocol**	**Anti-inflammatory activity and/or mechanism**	**Animal tested**	**Reference**
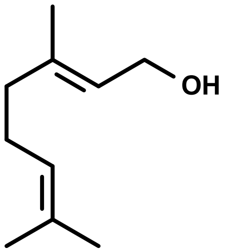 **Geraniol**	Lymphocyte proliferation assay; Cardiac allograft transplant model	Inhibition of lymphocyte proliferation; Prolonged graft survival	Rat	[81]
	Bacterial-induced inflammation	Reduced levels of inflammatory markers	Raw 264.7 macrophages	[82,83]
	LPS-induced NO and PGE2 production; LPS-induced COX-2 expression	Reduced iNOS mRNA expression; Reduced iNOS enzymatic activity; Reduced COX-2 mRNA expression; Activated PPARα and γ	Raw 264.7 macrophages	[84,85]
	Neutrophil activation	Reduced TNF-α-induced neutrophil adherence	Human	[87]
	LPS-induced NO production	Suppressed NO synthesis	Murine raw 264.7 macrophages	[88]
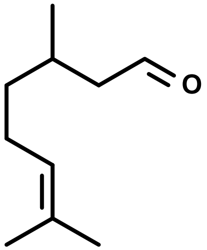 **Citronellal**	LPS-induced NO and PGE2 production; LPS-induced COX-2 expression	Reduced iNOS mRNA expression; Reduced iNOS enzymatic activity; Reduced COX-2 mRNA expression; Activated PPARα and γ	Raw 264.7 macrophages	[84,85]
	LPS-induced NO production	Suppressed NO synthesis	Murine raw 264.7 macrophages	[88]

Table S1. *Cont.*

	Experimental protocol	Anti-inflammatory activity and/or mechanism	Animal tested	Reference
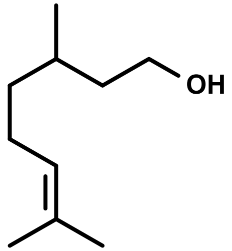 **Citronellol**	Neutrophil activation	Reduced TNF-α-induced neutrophil adherence	Human	[87]
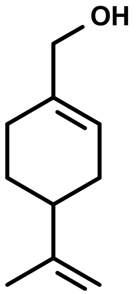 **Perillyl alcohol**	DMBA-initiated and TPA-promoted skin tumorigenesis	Inhibited ODC activity;<br>Reduced thymidine incorporation;<br>Inhibiton of RAS/Raf?ERK pathway	Mice	[91]
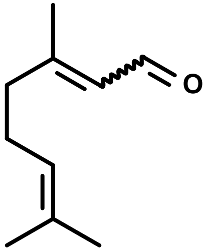 **Citral**	Neutrophil activation	Reduced TNF-α-induced neutrophil adherence	Human	[87]
	Paw edema ;<br>Carrageenan-induced leukocyte migration	Reduced paw edema and leukocyte migration	Rat	[95]
	LPS-induced NO production	Reduced NO production and iNOS expression;<br>Suppressed NF-*k*B activation	Raw 264.7 macrophages	[96]

Table S1. *Cont.*

	Experimental protocol	Anti-inflammatory activity and/or mechanism	Animal tested	Reference
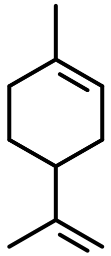 **Limonene**	LPS-induced NO and PGE2production	Inhibited NO and PGE2 production;<br>Reduced iNOS and COX-2 expression;<br>Reduced TNF-α, IL-1β, and IL-6 production	Raw 264.7 macrophages	[97]
	Eosinophilic leukemia HL-60 clone 15 cells	Suppressed ROS production and chemotaxis in p38 MAPK;<br>Reduced MCP-1 production via NF-*k*B activation	Human	[98]
	LPS-induced pleurisy	Reduced cell migration	Mice	[102]
	Epithelial buccal cells	Suppressed NO production;<br>Reduced IFN-γ an IL-4 production;<br>Suppressed IL-6 production;<br>Increased IL-10 production	Human;<br>macrophage	[103]
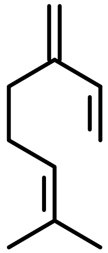 **Myrcene**	LPS-induced pleurisy	Reduced cell migration;<br>Suppressed NO production;<br>Reduced IFN-γ an IL-4 production	Mice	[102]

Table S1. *Cont.*

	**Experimental protocol**	**Anti-inflammatory activity and/or mechanism**	**Animal tested**	**Reference**
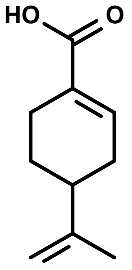 **Perillic acid**	Immuno assays	Immunostimulatory activity; Increased number of white blood cells and total antibody	Balb/c mice	[105]
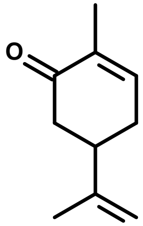 **Carvone**	Immuno assays	Immunostimulatory activity; Increased number of white blood cells and total antibody	Balb/c mice	[105]
	Neutrophil activation	Reduced TNF-α-induced neutrophil adherence	Human	[87]
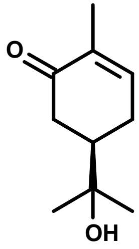 **Hydroxydihydrocarvone**	Carrageenan-induced paw edema; MPO activity	Reduced paw edema, MPO activity and neutrophil recruitment	Rat	[106]

Table S1. *Cont.*

	Experimental protocol	Anti-inflammatory activity and/or mechanism	Animal tested	Reference
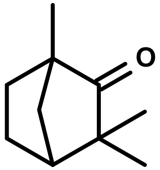 **Fenchone**	Carrageenan-induced paw edema	Reduced paw edema	Rat	[110]
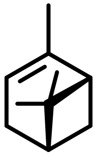 **α-Pinene**	Carrageenan-induced paw edema	Reduced paw edema; Reduced mechanical sensitization	Mice	[111]
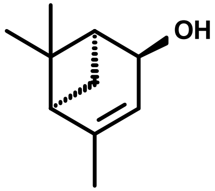 ***cis*-Verbenol**	Cerebral schemic-induced injury	Reduced pro-inflammatory cytokines expression	Rodent	[112]
